# Prevalence of fatigue and cognitive impairment after traumatic brain injury

**DOI:** 10.1371/journal.pone.0300910

**Published:** 2024-03-22

**Authors:** Traver J. Wright, Timothy R. Elliott, Kathleen M. Randolph, Richard B. Pyles, Brent E. Masel, Randall J. Urban, Melinda Sheffield-Moore

**Affiliations:** 1 Department of Internal Medicine, The University of Texas Medical Branch, Galveston, Texas, United States of America; 2 Department of Educational Psychology, Texas A&M University, College Station, Texas, United States of America; 3 Department of Pediatrics, The University of Texas Medical Branch, Galveston, Texas, United States of America; 4 Department of Neurology, The University of Texas Medical Branch, Galveston, Texas, United States of America; 5 Centre for Neuro Skills, Bakersfield, California, United States of America; St John’s University, UNITED STATES

## Abstract

**Background:**

Following traumatic brain injury (TBI) some patients develop lingering comorbid symptoms of fatigue and cognitive impairment. The mild cognitive impairment self-reported by patients is often not detected with neurocognitive tests making it difficult to determine how common and severe these symptoms are in individuals with a history of TBI. This study was conducted to determine the relative prevalence of fatigue and cognitive impairment in individuals with a history of TBI.

**Methods:**

The Fatigue and Altered Cognition Scale (FACs) digital questionnaire was used to assess self-reported fatigue and cognitive impairment. Adults aged 18–70 were digitally recruited for the online anonymous study. Eligible participants provided online consent, demographic data, information about lifetime TBI history, and completed the 20 item FACs questionnaire.

**Results:**

A total of 519 qualifying participants completed the online digital study which included 204 participants with a history of TBI of varied cause and severity and 315 with no history of TBI. FACs Total Score was significantly higher in the TBI group (57.7 ± 22.2) compared to non-TBI (39.5 ± 23.9; p<0.0001) indicating more fatigue and cognitive impairment. When stratified by TBI severity, FACs score was significantly higher for all severity including mild (53.9 ± 21.9, p<0.0001), moderate (54.8 ± 24.4, p<0.0001), and severe (59.7 ± 20.9, p<0.0001) TBI. Correlation analysis indicated that more severe TBI was associated with greater symptom severity (p<0.0001, r = 0.3165). Ancillary analysis also suggested that FACs scores may be elevated in participants with prior COVID-19 infection but no history of TBI.

**Conclusions:**

Adults with a history of even mild TBI report significantly greater fatigue and cognitive impairment than those with no history of TBI, and symptoms are more profound with greater TBI severity.

## Introduction

Traumatic brain injury (TBI) is the result of an injury to the head from an external force that may be fatal or results in decreased consciousness, amnesia, skull fracture, or neurological or neuropsychological abnormalities [1]. These injuries are common with an estimated 2.9 million TBI-related emergency department visits, hospitalizations, and deaths in the United States alone in 2014 [2]. This value underestimates the total TBI rate due to the large number of individuals that do not seek medical attention or are treated in non-hospital facilities [3]. Given the high incidence rate, TBI is a major cause of morbidity (including both short and long-term disability) and mortality, and results in a large financial burden [4, 5].

TBI may result from a variety of different injuries; the most common causes of nonfatal TBI-related hospitalization include unintentional falls, automotive accidents, other unintentional injuries, and violence [6]. Various instruments are used to classify the severity of TBI based primarily on assessment of responsiveness, loss of consciousness, and amnesia following injury [7]. Instruments designed for a clinical setting such as the Glasgow Coma Scale are useful for acute diagnosis of TBI by trained clinicians [8]. However, a large portion of TBIs go without acute clinical evaluation or treatment making it difficult to determine lifetime incidence rate of TBI among the general population [9]. The Ohio State University TBI Identification Method (OSU TBI-ID) was developed to standardize wording and capture lifetime TBI history in the general population [10].

Although rating scales vary, TBI severity is often classified as mild, moderate, or severe. Mild TBI is the most common and typically accounts for about 80% of TBI cases [11–13]. Greater TBI severity is generally associated with greater subsequent cognitive and behavioral symptoms. However, a number of confounding factors also affect TBI occurrence, symptom manifestation, and recovery [14]. Following TBI, functional outcomes (including fatigue and cognition) can be affected by various factors which include history of prior brain injuries, other concomitant trauma injuries, and age [15] heart or respiratory conditions and diabetes [16], substance use [17], psychiatric history and education level [18, 19]as well as vitamin D deficiency, sleep disturbance, and anxiety [20] The effects of various confounding factors and variable timeline of recovery have been previously studied and reviewed in detail [14, 21, 22] and the nuanced mechanisms of symptom manifestation and heterogeneity of confounding factors are beyond the direct scope of this study.

Given the heterogeneity in etiology, severity, individual medical history, and specific affected brain region, TBI severity does not necessarily reflect the pathophysiology, symptoms, treatment plan, or eventual recovery and outcome [23, 24]. Although most individuals with uncomplicated mild TBI fully recover in the first three months following injury, a subset of patients suffer long-term symptoms or disability following even mild TBI [25–27]. Among the most common of these lingering symptoms are fatigue and altered cognition (FAC) which is often described as “brain fog” [28, 29]. Past clinical and research experience led us to define brain injury associated fatigue and altered cognition (BIAFAC) as a clinical manifestation of these symptoms that may persist following TBI or even begin to manifest after the acute recovery period [30, 31]. Fatigue and cognitive impairment are distinct symptoms, but often co-manifest in TBI similar to Chronic Fatigue Syndrome [32] as well as other clinical conditions including Parkinson’s disease [33], multiple sclerosis [34], posttreatment Lyme disease syndrome [35], and following acute recovery from infectious diseases including COVID-19 [36].

Although persistent FAC symptoms are known to develop in a subset of individuals following TBI, it is not clear how the occurrence and severity of these symptoms vary in the general population, among individuals with a range of TBI severity, or following other clinical conditions. In order to study BIAFAC and other similarly manifesting clinical conditions, we developed the Fatigue and Altered Cognition Scale (FACs) as a sensitive and specific digital questionnaire to evaluate self-reported comorbid symptoms of fatigue and cognitive impairment [37]. The FACs was validated by comparing responses from research participants with a history of TBI to those without a TBI history. Here, we present an ancillary analysis of that questionnaire development data to describe and compare the occurrence of FAC symptoms within TBI and non-TBI populations.

## Methods

### Recruitment and participation

This study was approved by the Institutional Review Boards at Texas A&M University (IRB# 2021–0836) and the University of Texas Medical Branch (IRB# 21–0182). The results presented here are a substantive analysis of data used in the development of the FACs questionnaire. Adults ranging from 18 to 70 years of age were recruited by providing research team contact information using social media, university listservs, and researchmatch.org. ResearchMatch is a national health volunteer registry that was created by several academic institutions and supported by the U.S. National Institutes of Health as part of the Clinical Translational Science Award (CTSA) program. ResearchMatch has a large population of volunteers who have pre-consented to be contacted by researchers about health studies for which they may be eligible. In order to collect adequate representative data from both populations, ResearchMatch recruitment included both non-targeted recruitment of individuals that did not identify a history of TBI when registering, as well as targeted recruitment efforts for subjects that reported a history of TBI. Interested individuals that contacted the research team were provided individual anonymous links to access the questionnaire. Participants who initiated the questionnaire were digitally screened and consented as approved by IRB. The study was granted an IRB waiver of written consent due to anonymity and minimal risk of harm. Participants that were eligible upon screening, provided consent, and completed the full questionnaire were included in analysis. For the purposes of this study, subjects were grouped based on a self-reported history of TBI during screening. All data presented in this study was collected from August 27, 2021 to December 7, 2021.

### FACs questionnaire

A full description of the questionnaire and its development has been previously published [37]. In brief, the research team created 20 relevant questions based on clinical experience and existing literature for participants to self-assess fatigue and brain fog (see S1 File for question flow). The digital online questionnaire was developed using Qualtrics (Qualtrics International Inc. Seattle, WA, USA), for participants to self-administer on various digital devices including either phone, computer, or tablet. Response for each question was provided using an electronic visual analog response scale (eVAS) as an efficient measure of self-reported symptoms. This visual scale bar allowed subjects to score their agreement to each question along a continuous linear sliding scale from “not at all” (0) to “extremely” (100) without confirmation of the scored number (see S1 File for visual example). Participants were asked to consider the last two weeks in their response to each question. After completing the questionnaire, symptomatic patients were asked, “How long after your last brain injury did you start to experience any of these problems?” with potential answers of “Less than 6 months”, “6 months to 1 year”, “1 year to 5 years”, or “More than 5 years”. Participants then completed a modified version of the previously validated Ohio State University TBI Identification Method (OSU TBI-ID) to collect further information about lifetime history regarding the type and severity of TBI [10]. As previously reported, the median time to complete the questionnaire for non-TBI individuals was 3.7 minutes, and TBI individuals took a median of 6.2 minutes due to additional TBI-related questions [37].

### Quantifying TBI severity

In order to compare symptoms across a range of TBI severity, subjects were stratified into groups based on self-reported loss of consciousness (LOC) from their most severe head or neck injury as mild TBI (TBI with altered consciousness or LOC < 30 minutes), moderate TBI (LOC of 30 minutes to 24 hours), or severe TBI (LOC > 24 hours) [4, 38]. As an exploratory measure, TBI history responses collected from the digitized OSU TBI-ID were also used retrospectively to provide a quantitative assessment of TBI severity with greater resolution than a general classification. Select responses were weighted and scaled to numerical values and tallied to quantify brain injury severity as outlined below. Given the outlined scoring, participants could have a Severity Ranking score ranging from 0 (no TBI) to 9 (TBI with LOC > 24 hours, hospitalization following injury to head or neck, and a history of repeated impacts to the head). Although somewhat arbitrary, this exploratory ranking score provides a simple quantification of TBI severity based on retrospective recall of TBI history using the OSU TBI-ID.

Q1: Have you had a TBI/concussion?

    No            0

    Yes           1

Q2: In your lifetime, have you ever been hospitalized or treated in an emergency room following an injury to your head or neck?

    No            0

    Yes           2

Q3: Were you knocked out or did you lose consciousness (LOC)?

    Over 24 hours            4

    30 minutes to 24 hours         3

    Less than 30 minutes        2

    No                 0

    Q3b: (If “No” on Q3 above) Were you dazed or did you have a gap in your memory from the injury?

      Yes           1

      No            0

Q4: Have you ever had a period of time in which you experienced multiple, repeated impacts to your head (e.g. history of abuse, contact sports, military duty)?

    Yes           2

    No            0

### COVID-19

Because this study was conducted during the COVID-19 pandemic, participants were also asked if they had been diagnosed with COVID-19. Of the 519 participants, 64 (12.3%) reported having had a COVID-19 infection at any point prior to their participation. An ancillary exploratory analysis was conducted and presented here to explore potential effects of COVID-19 infection on FACs score. Given the profound effect of reported TBI history on FACs score, the potential impact of COVID-19 was assessed separately within TBI and non-TBI groups.

### Statistics

Statistical analysis was done using GraphPad Prism 10.0.0 (GraphPad Software, LLC) with p<0.05 as the threshold for statistical significance. Fisher’s exact test was used to compare the occurrence rate of discrete variables between groups. Unpaired t-tests were used for direct comparison of mean values between two groups. Pearson correlation was used to explore associations between continuous variables. One-way ANOVA was used for multiple groupwise comparisons followed by post-hoc Tukey’s multiple comparison. The ability of the FACs questionnaire Total Score to differentiate TBI and non-TBI reporting individuals was assessed by receiver operating characteristic (ROC) curve; accuracy was estimated with total area under the curve (AUC). Even distribution of age was assessed with Chi-square goodness of fit using 9.792 expected observations for each year of age in the study (519 subjects/ 53 years included age). Five individuals in the non-TBI group identified as nonbinary sex and were excluded from sex-based comparisons leaving 310 in the non-TBI group and 204 in the TBI group.

## Results

### Study demographics

A total of 776 participants initially responded to the recruitment efforts for the FACs questionnaire. Eight individuals declined consent and 35 indicated that they were not able to provide consent. Individuals that did not provide consent were immediately exited from the study without collecting further information. An additional 214 were excluded from analysis because they consented and began the questionnaire but did not complete all questions. A total of 519 qualified participants completed the study and were included in final analysis.

Given options to self-identify as “Male”, “Female”, or “Not Listed: Please Specify”, five individuals identified as either nonbinary (n = 3), no gender (n = 1), or trans male (n = 1). Given insufficient numbers for groupwise comparison, individuals that did not primarily identify as either male or female were not included in sex-based comparative analysis. All qualified individuals were included in non sex-based analysis. A significantly greater number of females chose to participate (P<0.0001), with 379 qualifying females (73.7%) and 135 qualifying males (26.3%). Of the 519 qualifying participants, 204 reported a history of TBI (59 m, 145 f) and 315 reported no history of TBI (76 m, 234 f). Male subjects were on average more likely to report a history of TBI (Male: 59/135, 43.7%; Female: 145/379, 38.3%), but differences were not statistically significant (p = 0.305; OR = 1.253).

The average age of subjects reporting a history of TBI (42.0 ± 15.1 years) was significantly older than those reporting no history of TBI (39.1 ± 16.9 years; p = 0.0454) although the age difference was slight (Table 1). Based on goodness of fit analysis, participation was not evenly distributed across the included age range of 18 to 70 years of age (p<0.0001) with individuals under 25 years of age disproportionately represented (S1 Fig in S1 File).

**Table 1:** Demographic distribution of participants in the current study. A significantly greater number of females participated than males. The average age of participants in the traumatic brain injury (TBI) group was significantly older than the non-TBI group.

		Total	non-TBI	TBI	*P*
Qualified Participants		519	315	204	
Sex					<0.0001
	Male	135	76	59	
	Female	379	234	145	
	Sex Not Reported	5	5	0	
Age		40.2 ± 16.3	39.1 ± 16.9	42.0 ± 15.1	0.0454

### TBI occurrence

The reported occurrence, cause, and severity of TBI varied among study participants (Table 2). It is important to note that some participants reported multiple TBI, and are therefore included more than once in TBI Cause. As a result, the summative reporting totaled more than 100%. Participants with a history of TBI indicated that in their lifetime they have had an injury to the head or neck resulting from falls (76%), car crashes (54.9%), violence (21.1%), or proximity to an explosion (5.9%).

**Table 2:** Traumatic brain injury (TBI) related reported history for participants completing the Fatigue and Altered Cognition Scale (FACs) questionnaire. TBI causes are categorized based on data collected with the digitized OSU TBI-ID. Some individuals reported more than one TBI and are therefore represented in more than one category for TBI Cause.

		Number	Portion of TBI (%)
Reported no TBI		315	
Reported TBI		204	
TBI Cause ^1^			
	Fall	155	76.0%
	Car Crash	112	54.9%
	Fight	43	21.1%
	Explosion	12	5.9%
TBI Loss of Consciousness			
	No Loss of Consciousness	74	36.3%
	Less than 30 min.	80	39.2%
	30 min. - 24 hours	24	11.8%
	Over 24 hours	26	12.7%
TBI Hospitalization ^2^		151	74.0%

1 some participants have experienced more than one TBI

2 subjects were asked, "In your lifetime, have you ever been hospitalized or treated in an emergency room following an injury to your head or neck? "

Among participants reporting TBI, 63.7% reported having experienced some LOC resulting from TBI. Based on LOC classification of TBI severity for an individual’s most severe injury to the head or neck, 75.5% were classified mild TBI, 11.8% were classified moderate TBI, and 12.7% were classified severe TBI. Seventy-four percent of participants reporting a history of TBI indicated that at some point in their life they had been hospitalized or treated in an emergency room following an injury to the head or neck.

### FAC symptoms—TBI and non-TBI populations

**Fig 1:**
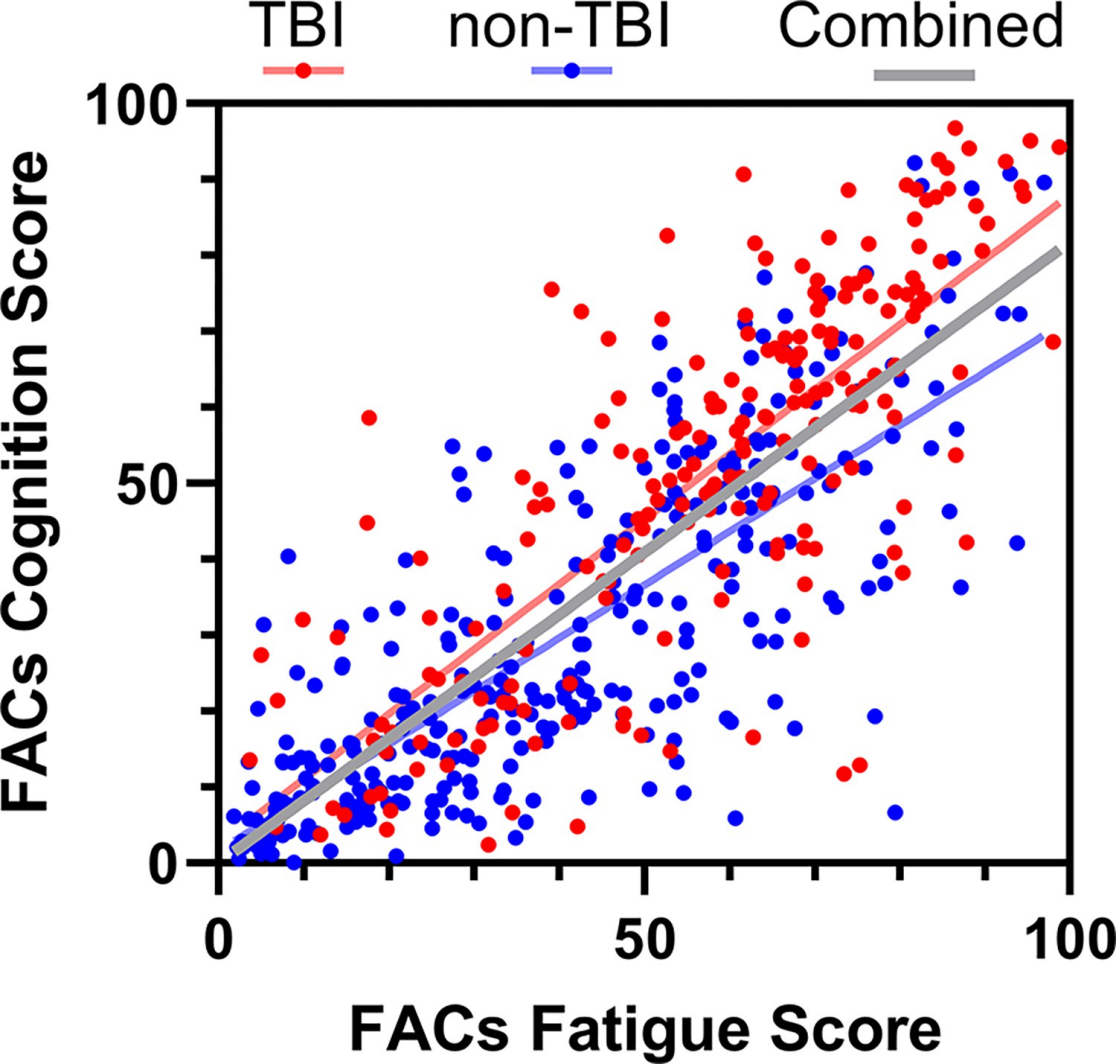
For adults taking the online Fatigue and Altered Cognition Scale (FACs) questionnaire there was strong correlation between the two subtests assessing fatigue and cognition for participants reporting a history of traumatic brain injury (TBI; p<0.0001, r = 0.7782), no reported history of TBI (non-TBI; p<0.0001, r = 0.7828), and combined (p<0.0001, r = 0.8089).

Symptoms of fatigue and cognitive impairment are often comorbid among individuals with a variety of clinical conditions. There was a strong correlation between Fatigue and Cognition subtest scores of the FACs questionnaire in both TBI (Fig 1; p<0.0001, r = 0.7782) and non-TBI groups (p<0.0001, r = 0.7828), as well as among all combined participants (p<0.0001, r = 0.8089).

**Fig 2:**
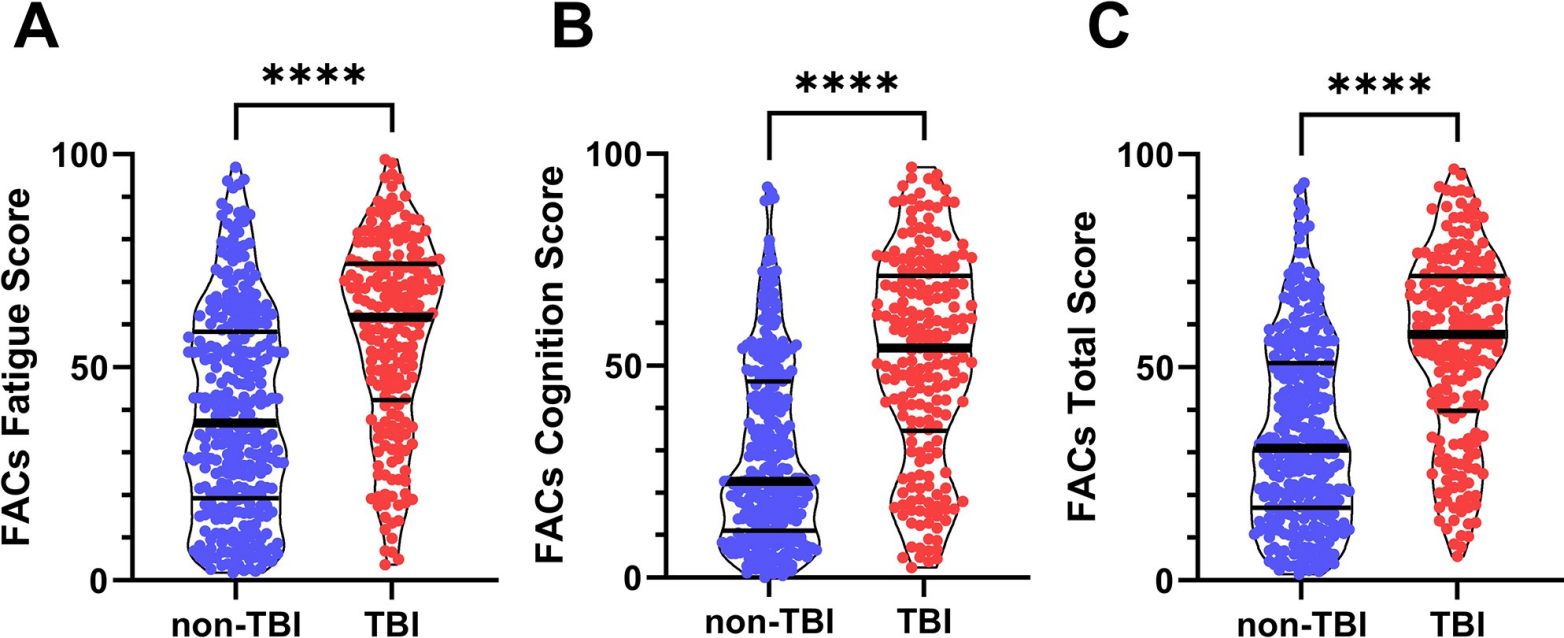
Fatigue and Altered Cognition Scale (FACs) questionnaire scores for participants with a history of traumatic brain injury (TBI) and those without. Participants with a self-reported history of TBI reported significantly greater symptom severity (higher FACs score) for (**A**) Fatigue (p<0.0001), (**B**) Cognition (p<0.0001), and (**C**) Total Score (p<0.0001).

Subjects reporting a history of TBI had significantly higher FACs questionnaire scores. The Fatigue scale score was significantly greater in the TBI group (57.7 ± 22.2) than non-TBI (39.5 ± 23.9) indicating greater self-reported fatigue associated with a history of TBI (Mean Difference 18.2; 95%CI [14.1, 22.3]; p < 0.0001; Fig 2). The Cognition scale score was also significantly greater in the TBI group (51.8 ± 24.5) than non-TBI (29.3 ± 21.4) indicating greater self-reported symptoms of cognitive impairment in the TBI group (Mean Difference 22.6; 95% CI [18.6, 26.6]; p < 0.0001). Combined FACs Total Score was also significantly greater in the TBI group (54.8 ± 22.0) than non-TBI (34.4 ± 21.4) (Mean Difference 20.4; 95% CI [16.6, 24.2]; p < 0.0001).

**Fig 3:**
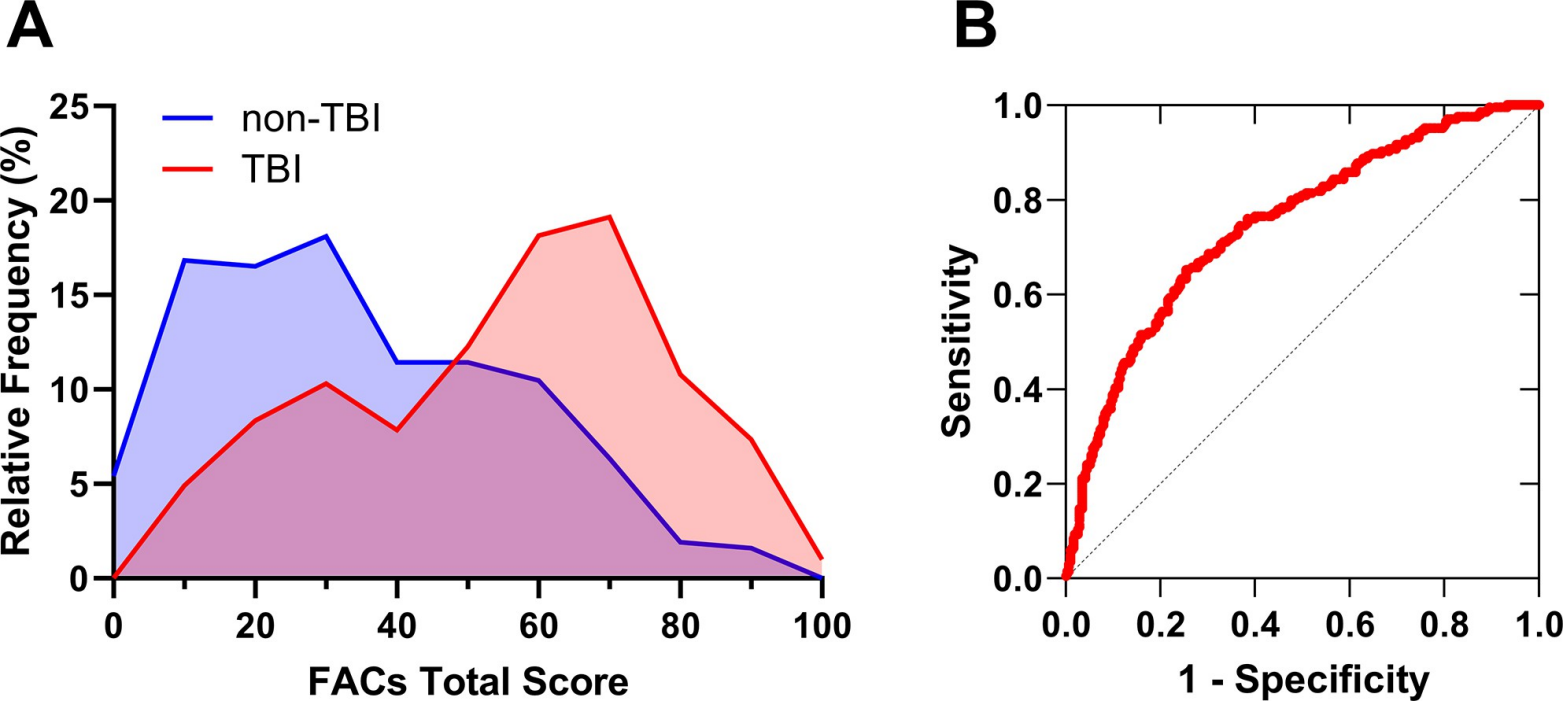
The Fatigue and Altered Cognition Scale (FACs) questionnaire can distinguish groups with and without a history of traumatic brain injury (TBI). (**A**) Frequency distribution histogram (binned in 10 point intervals) of FACs Total Score for subjects reporting a history of TBI or no history of TBI. (**B**) Receiver operating characteristic (ROC) curve suggests the FACs questionnaire adequately differentiated individuals with a history of TBI from non-TBI (p<0.0001; ROC AUC = 0.7466).

Given the distinct distribution of FACs Total Score between TBI and non-TBI populations (Fig 3A), we assessed the ability of the FACs questionnaire to distinguish between the two groups using a receiver operating characteristic (ROC) curve (Fig 3B). Total area under the resulting ROC curve (0.7466) suggested that FACs Total Score is a significant indicator of a history of TBI (p<0.0001).

### TBI severity and FAC symptoms

**Fig 4:**
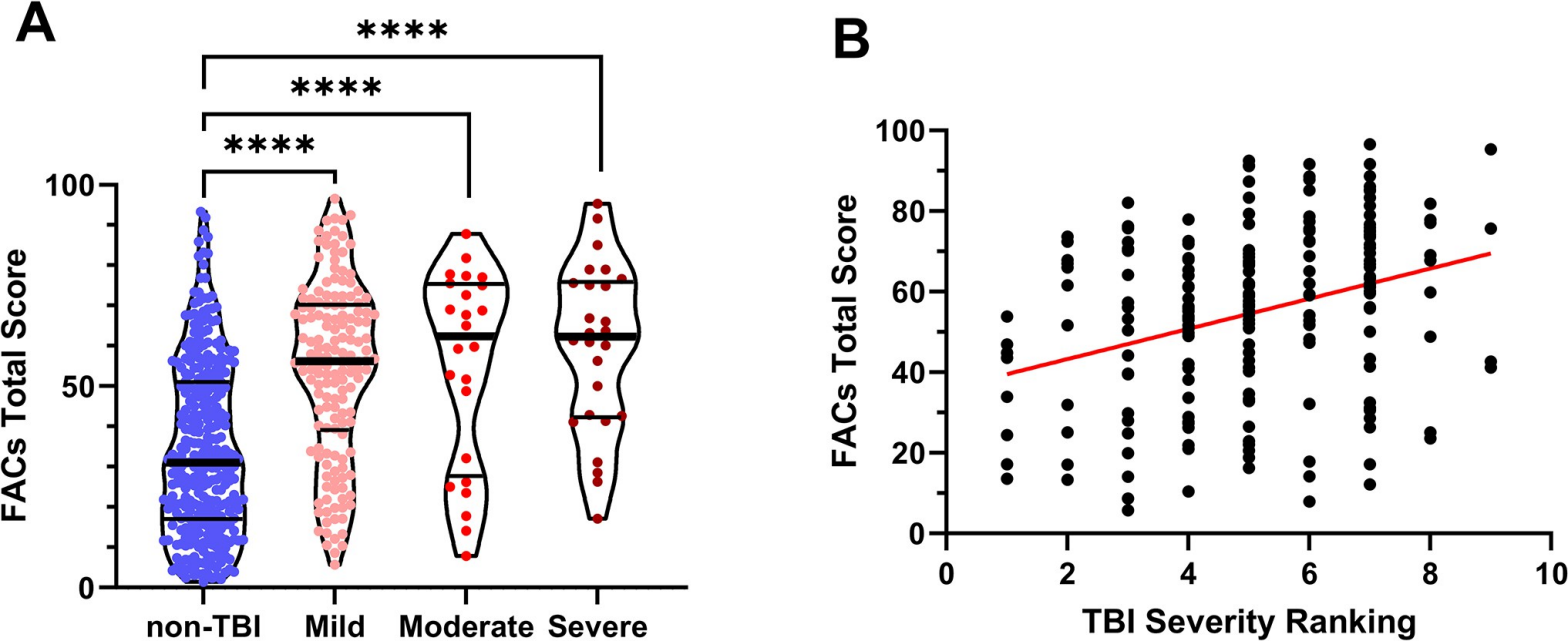
Impact of traumatic brain injury (TBI) severity on Fatigue and Altered Cognition Scale (FACs) score. **A)** Participants with no history of TBI had significantly lower FACs Total Score than those reporting a history of TBI ranked as Mild, Moderate, or Severe (each p<0.0001). There were no significant groupwise differences among TBI groups. **B**) For individuals reporting a history of TBI, FACs Total Score was positively correlated with post-hoc ranking of TBI severity based on self-reported injury outcomes (r = 0.3165, p<0.0001).

In order to determine if FACs score was influenced by TBI severity, participants were classified based on reported LOC as mild, moderate, or severe TBI. The FACs Total Score was significantly different between groups (ANOVA; p<0.0001). In multiple comparison testing, there was no significant difference among the three TBI severity groups. However, the non-TBI group (34.4 ± 21.4) was significantly lower than each TBI severity group including mild (53.9 ± 21.9)(Mean Difference -19.5; 95% CI [-25.0, -14.1]; p<0.0001), moderate (54.8 ± 24.4)(Mean Difference -20.4; 95% CI [-32.2, -8.6]; p<0.0001), and severe (59.7 ± 20.9)(Mean Difference -25.3; 95% CI [-36.7, -13.9]; p<0.0001) (Fig 4A). Weighted scoring of TBI questions was used to provide a greater resolution scoring of TBI severity (see Methods: Quantifying TBI Severity). Based on this higher resolution ranking, TBI severity was significantly correlated with FACs total score (p<0.0001, r = 0.3165; Fig 4B).

### FAC symptom timing and recovery after TBI

To assess timing of symptom presentation after TBI, symptomatic TBI participants were asked how long it was after their last brain injury before they began to experience symptoms. Among the 199 qualifying participants, 123 (61.8%) reported experiencing symptoms in less than 6 months, 18 (9.0%) in 6 months to 1 year, 28 (14.1%) in 1 year to 5 years, and 30 (15.1%) in more than 5 years.

**Fig 5:**
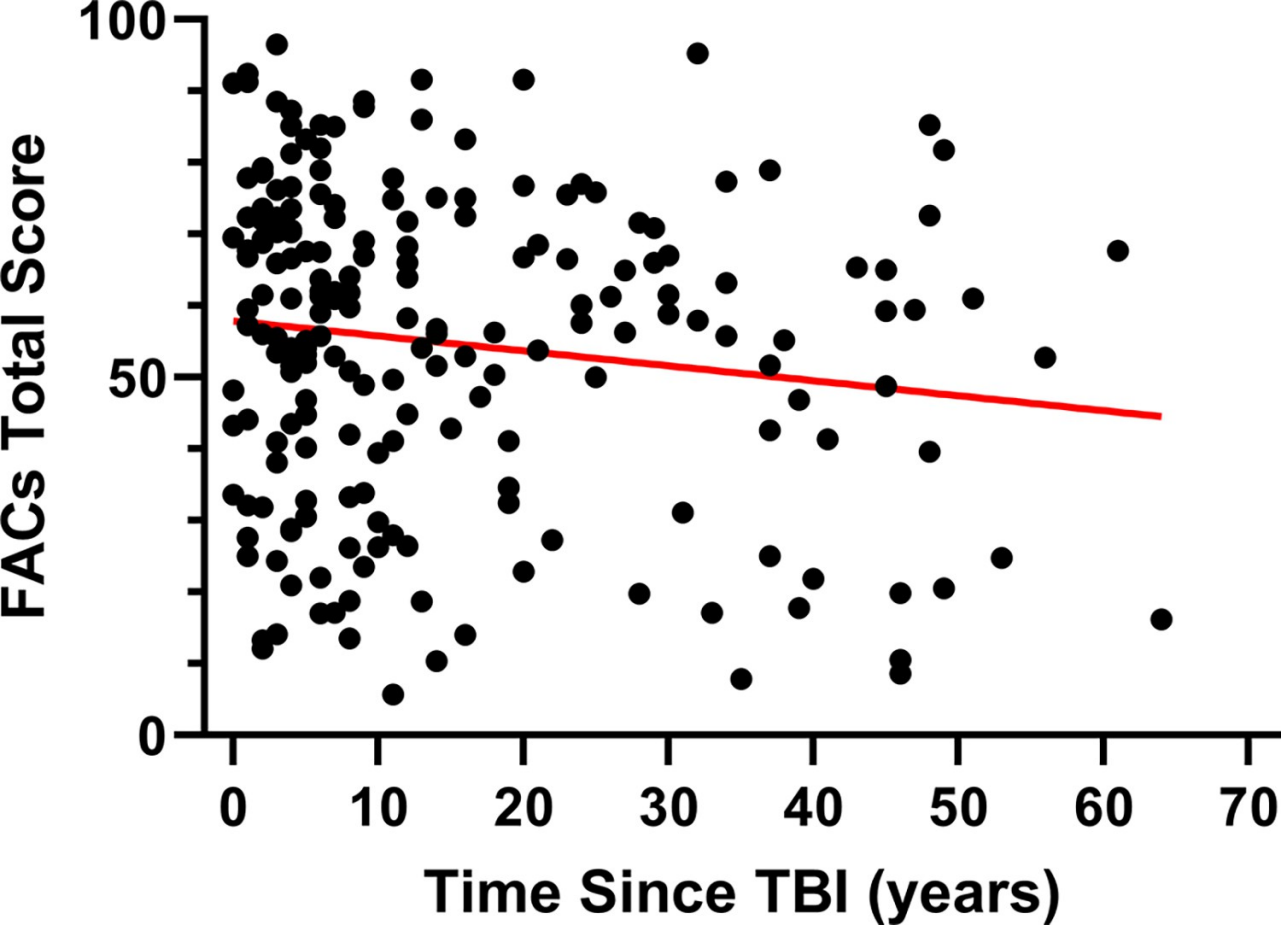
The influence of recovery time after traumatic brain injury (TBI) on symptoms assessed by the Fatigue and Altered Cognition Scale (FACs) questionnaire. Greater recovery time after TBI was associated with modest improvement of FACs Total Score (p = 0.043, r = -0.1418).

Pearson correlation was used to determine if FACs Total Score was influenced by recovery time following TBI (Fig 5). The time since injury for subjects reporting a history of TBI ranged from 0 to 64 years (Ave: 15.0 ± 14.9). There was a modest but significant negative correlation between FACs Total Score and time since TBI (p = 0.043, r = -0.1418).

### COVID and FACs

**Fig 6:**
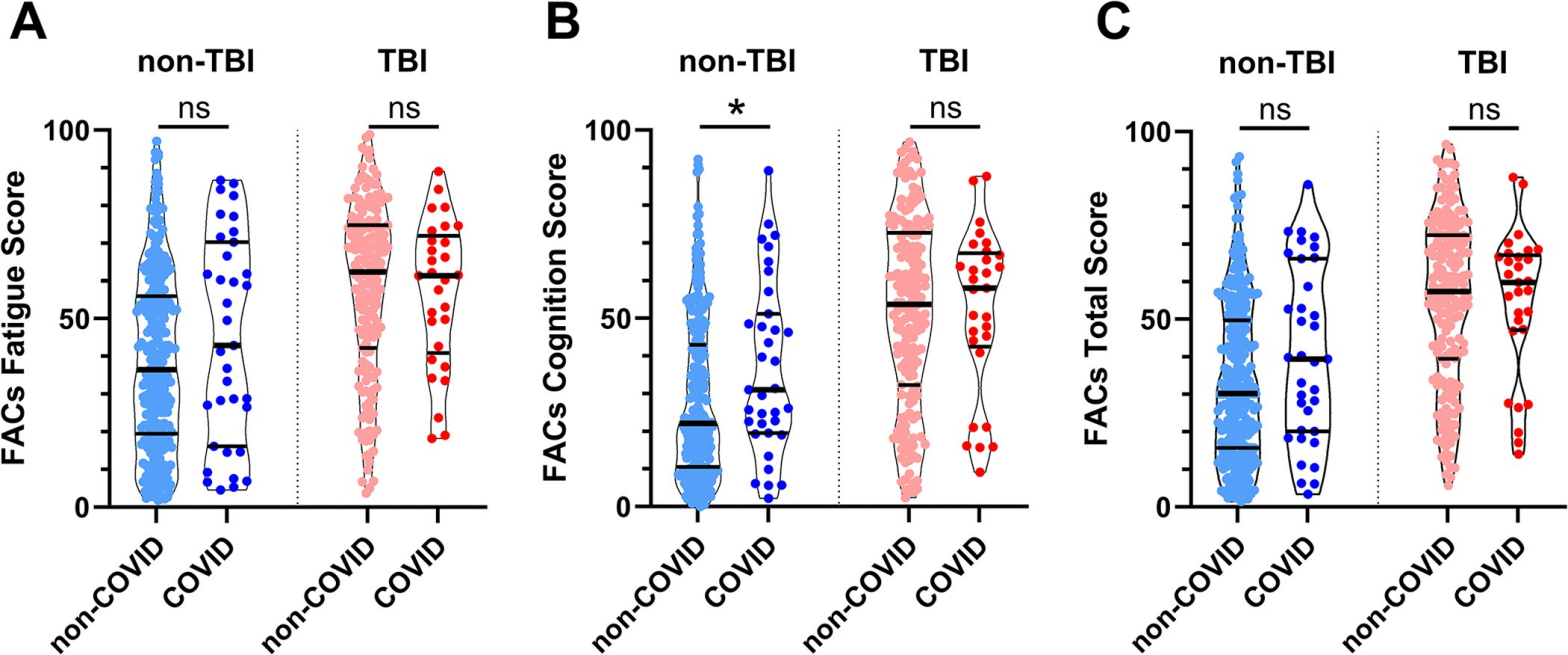
Fatigue and Altered Cognition Scale (FACs) questionnaire scores for participants with or without prior history of traumatic brain injury (TBI) and COVID-19 infection. A history of COVID-19 infection did not influence average FACs scores for subjects with a prior history of TBI. For participants with no reported history of TBI, COVID-19 infection was associated with significantly higher FACs Cognition Score (p = 0.029) and non-significant higher average scores for Fatigue Score (p = 0.184) and Total Score (p = 0.066).

In an exploratory ancillary analysis, reported history of COVID infection at any point prior to participation was also assessed for effect on FACs score. Potential effects of COVID diagnosis were assessed independently within the TBI and non-TBI groups using unpaired t-tests to account for the overwhelming effect of TBI. Of the 204 TBI subjects, 29 reported a COVID diagnosis and 175 reported no COVID diagnosis. For subjects with a history of TBI, COVID diagnosis did not affect average Total Score (Mean Difference -0.2; 95% CI [-9.0, 8.5]; p = 0.957), Fatigue score (Mean Difference -1.0; 95% CI [-9.8, 7.8]; p = 0.829), or Cognition score (Mean Difference 0.5; 95% CI [-9.2, 10.2]; p = 0.921; Fig 6). Of the 315 non-TBI subjects, 35 reported a COVID diagnosis and 280 reported no COVID diagnosis. For subjects without a history of TBI, Fatigue Score was not significantly different between groups (Mean Difference 5.7; 95% CI [-2.7, 14.1]; p = 0.184), however, Cognition Score was significantly higher in subjects with prior COVID diagnosis (Mean Difference 8.4; 95% CI [0.9, 15.9]; p = 0.029). The average Total Score trended higher for subjects with a COVID diagnosis but was not statistically significant (Mean Difference 7.0; 95% CI [-0.5, 14.6]; p = 0.066).

## Discussion

### Demographics and participation

Women were significantly more likely to participate in the current online anonymous study, constituting 73.7% of participants (Table 1). This unequal sex distribution is consistent with the greater willingness of females to volunteer in health research surveys [39] and the demographic composition of ResearchMatch.org (a major source of recruitment for the current study) which reports volunteer demographics of 69.8% female and 30.2% male (www.researchmatch.org/volunteers/).

Given the increased likelihood of receiving a TBI with a greater number of years lived, it is not surprising that the TBI group skewed significantly older (42.0 ± 15.1 years) than the non-TBI group (39.1 ± 16.9 years; p = 0.0454; Table 1). Although the TBI group was significantly older than the non-TBI group, this age disparity was not a driving factor in the groupwise difference in FACs Total Score given that FACs score was negatively correlated with age (S2 Fig in S1 File; slope = -0.2456; R^2^ = 0.0282; p = 0.0001). Although lower reported fatigue with age may be counterintuitive, this is consistent with results from other recent studies of general and task-based fatigue [40, 41].

The population sampled here is similar to previously reported distributions of TBI cause and severity. The most common reported causes of TBI in the current study included falls and vehicle accidents (Table 2) which aligns with established trends [6, 12, 13]. Mild TBI generally accounts for approximately 80% of TBI, with Moderate and Severe TBI each constituting approximately 10% [11]. This is similar to the distribution of TBI severity seen in the current study with 75.5% Mild TBI, 11.8% Moderate TBI, and 12.7% Severe TBI.

### FACs and TBI

Following TBI of all severity, a subset of individuals report “brain fog” as a symptom which includes perceived fatigue and cognitive impairment [28, 29]. Individual reports of fatigue are inherently subjective, and the associated cognitive impairment may be subtle and perceptible to individuals through effects on daily life without being detected by standard neuropsychological testing [42]. Although symptoms of fatigue and cognitive impairment are distinct and warrant individual testing, they often co-manifest [32, 43]. In the current study, strong correlation between FACs subtest scores for Fatigue and Cognition in both TBI and non-TBI groups suggest strong overlap in symptom presentation in both healthy and clinical populations (Fig 1).

In the current study, participants with a history of TBI scored significantly higher (greater symptom severity) on FACs Total Score and reported significantly more symptoms in individual subtests for both Fatigue and Cognition (Fig 2). The sharp distinction in FACs score distribution between TBI and non-TBI populations is surprising considering that a) conditions other than TBI can trigger similar symptoms, and b) not all individuals who suffer from TBI (including a wide spectrum of cause and severity) develop symptoms. Given the myriad conditions that may result in fatigue and brain fog, the FACs questionnaire proved a remarkably good indicator of a history of TBI. While the FACs questionnaire is certainly not diagnostic of TBI, the strong association between TBI history and FAC symptoms demonstrated by the ROC curve (Fig 3B) suggests that these symptoms may be more common following TBI of all cause and severity than currently appreciated.

Individuals with a history of even mild TBI report activity limitations and lower life satisfaction, and these negative outcomes worsen with increased TBI severity [3]. Along with prior studies, our current results indicate some correlation between TBI severity and greater symptoms. However, ranking of TBI severity is not necessarily predictive of self-reported symptoms and quality of life [24]. Assessing TBI severity, outcome, and treatment options may be much more nuanced [23]. Based on groupwise comparison of mild, moderate, and severe TBI (assessed by LOC duration), even mild TBI was associated with significantly worse symptoms compared to the non-TBI group (Fig 4A). Based on this crude classification of TBI severity, there was no distinction in symptom severity within the various TBI groups. Investigating TBI severity at higher resolution using weighted scoring of participant responses to the modified digital OSU TBI-ID did reveal a significant link between greater TBI severity and worse outcome (Fig 4B).

### Symptom onset and recovery

It is not clear why some individuals develop FAC symptoms following TBI while others do not, or why the onset of those symptoms is variable. Although 61.8% of symptomatic participants in the current study reported symptoms within 6 months of the last TBI, nearly 30% reported that symptoms first began a year or more after injury with over 15% reporting symptoms more than 5 years later. FAC-like symptoms can manifest from a variety of conditions, and delayed symptoms reported by some may be unrelated to the prior TBI. However, the strong links between individuals with a history of TBI and the presence of FAC symptoms suggest that TBI may initiate a delayed onset of symptoms or predispose and sensitize individuals to later neuroinflammatory triggers. Evidence suggests that cumulative systemic inflammatory burden can influence neuroinflammation, cognition, and fatigue [44–48]. Eventual but delayed onset of FAC symptoms may reflect the TBI as one of multiple cumulative neuroinflammatory triggers leading to eventual symptom progression.

Longitudinal studies demonstrate an initial TBI recovery period. Although recovery is most rapid in the first six months after injury, most mild or moderately-severe TBI patients report lingering but progressive improvement in symptoms for at least 5 years post injury [24]. In the current study we detected a small but significant trend for improvement in FACs total score associated with increased time post-injury (Fig 5). The slight but significant negative correlation between TBI recovery time and FACs Total Score suggests that there may be some modest symptom improvement over time. However, given the similar trend associated with age in general (S2 Fig in S1 File), this trend is not convincingly associated with recovery time in our cross-sectional study. Individuals with lingering symptoms following the initial recovery period may not see symptom relief without proper assessment, treatment, and monitoring. In addition, the onset of symptoms may not always occur immediately after TBI.

### FACs threshold

We suggest that the groupwise FACs score distribution intercepts (Fig 3A) and the overlapping optimization of individualized ROC sensitivity and specificity plots (S3 Fig in S1 File) provide justification for designating a FACs total score of 50 as a threshold indicating meaningful symptom severity. Only 25.4% of subjects without a history of TBI scored above 50 on the FACs Total Score, while 64.7% of subjects with a history of TBI scored greater than 50. The study population undoubtedly included individuals with confounding clinical conditions and no history of TBI as well as individuals with a history of TBI and low FACs score. The FACs was surprisingly adept at identifying individuals with TBI despite these confounding factors. Coupled with a history of TBI, a FACs Total Score greater than 50 could warrant further clinical investigation (including testing of endocrine function) and possible treatment to relieve symptoms. A similar scoring threshold may also be relevant for other conditions that manifest with overlapping symptoms.

### FACs and PASC

Because this study was ongoing during the emerging COVID-19 pandemic, it provided an opportunity to capture potentially relevant data related to COVID-19 infection and subsequent overlapping post-acute sequelae of COVID-19 (PASC) symptoms. Fatigue and brain fog are two of the most common symptoms associated with both acute SARS-CoV-2 infection and lingering PASC symptoms [49]. PASC-associated cognitive symptoms and fatigue (low motivation or energy) are associated with neuroinflammation [50], suggesting potential overlapping mechanisms. Within the group of subjects that reported a history of TBI, average FACs scores were not different between participants with prior SARS-CoV-2 infection and those without (Fig 6). However, within the group of subjects with no reported history of TBI, there were trends distinguishing COVID-19 patients from those without. If these disparate conditions that may include TBI, PASC, and other conditions that manifest with similar neurologic symptom (including fatigue and brain fog) and share common mechanistic triggers (e.g. neuroinflammation), the symptoms may not be additive and cumulative, but a more binary response to a neuroinflammatory threshold. In this way, existing neurologic symptoms from prior maladies may mask effects of newly acquired conditions.

### Study limitations

The use of questionnaires to assess symptoms is necessarily subjective, relying on self-reporting from individuals which may introduce selection bias for individuals willing or motivated to complete the questionnaire. This study was not designed to record and account for the myriad pre-existing and concomitant conditions which can affect occurrence, etiology, and recovery following TBI. Although there are numerous confounding factors to TBI sequelae, adequate sample size helps buffer distribution of factors among groups not specifically linked with TBI. A key strength of the FACs questionnaire is the ability to efficiently survey and score responses from a large number of individuals. However, unsupervised screening relies on accurate self-reporting. Instruments such as the Glasgow Coma Scale are useful for contemporaneous diagnosis of TBI [8], but retrospective self-assessment of TBI relies on consistent definition of TBI and ranking criteria which can lead to inconsistencies in TBI self-reporting [3, 5, 9, 38, 51]. We incorporated a modified digital version of the OSU TBI-ID for standardized language to query participants about lifetime history of TBI [10].

Study enrollment was not controlled to capture a demographic distribution that represents the overall population. Uneven demographic representation of factors including age, and sex are evident; younger individuals are over-represented in the study which may be related to the online format of recruitment and data collection self-selecting for a younger demographic with greater online presence. Younger individuals are more likely to participate in health research surveys using an online format [39]. Although the demographic distribution of the current study does not represent the overall population, it provides important information regarding the occurrence and severity of FAC symptoms associated with TBI. It is important to note that because study recruitment efforts included targeted recruitment of individuals with a history of TBI, this data is not suitable to estimate the incidence rate of TBI in the general population. However, conclusions within the TBI group and comparisons between TBI and non-TBI subjects are justified.

### Conclusions

Individuals with a history of TBI of all severity (including mild TBI) report significantly greater symptoms of fatigue and cognitive impairment than those without a history of TBI. The FACs is a sensitive and specific instrument to efficiently assess clustered symptoms of fatigue and altered cognition (brain fog) common to a variety of clinical conditions. It can be used to quantify self-reported symptoms and monitor symptom progression for clinical and research purposes. We feel our data suggests that a FACs Total Score >50 represents a threshold for meaningful symptom impact.

## Supporting information

S1 File (PDF)
