# Supplementary material for: Prevalence of fatigue and cognitive impairment after traumatic brain injury
Source: PLoS One. 2024 Mar 22;19(3):e0300910. doi: 10.1371/journal.pone.0300910 (PMC10959386; doi:10.1371/journal.pone.0300910)
Supplement: S1 File — (PDF) [file pone.0300910.s001.pdf]

Supplemental material for:

Prevalence of fatigue and cognitive impairment after traumatic brain injury

Authors:

Traver J. Wright[1]; Timothy R. Elliott[2]; Kathleen M. Randolph[1]; Richard B. Pyles[3]; Brent E. Masel[4,5]; Randall J. Urban[1]; Melinda Sheffield-Moore[1]

Affiliations:

[1] Department of Internal Medicine, The University of Texas Medical Branch; Galveston, Texas, 77555, USA.
[2] Department of Educational Psychology, Texas A&M University, College Station, Texas; USA
[3] Department of Pediatrics, The University of Texas Medical Branch; Galveston, Texas, 77555, USA.
[4] Department of Neurology, The University of Texas Medical Branch; Galveston, Texas, 77555, USA
[5] Centre for Neuro Skills; Bakersfield, California, 93313, USA

Corresponding Author:

Traver Wright
traywright@gmail.com

This file includes:

Figures S1-S3

Survey Question Flow: Screening, Demographics, and Study Questions For the Fatigue and Altered Cognition Scale (FACs)

SUPPLEMENTAL MATERIALS

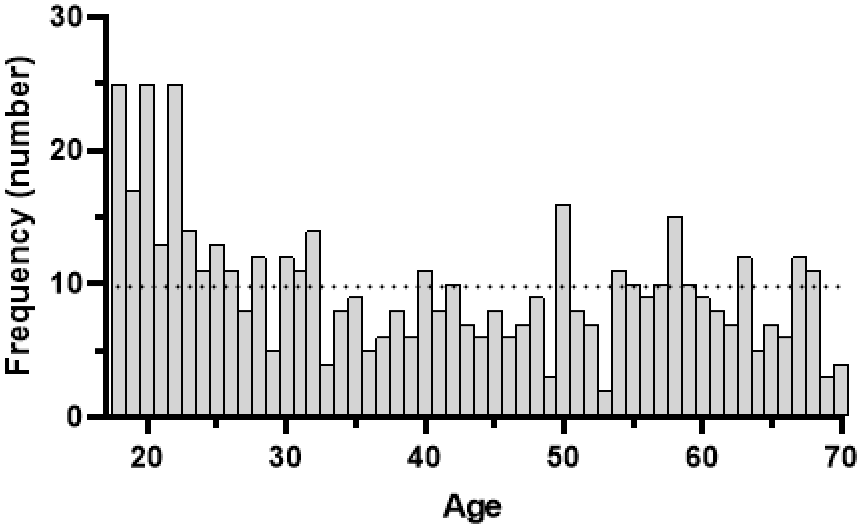

Fig. S1 Age distribution of adults (18-70 years) participating in an online study of fatigue and cognitive impairment in subjects with and without a history of traumatic brain injury. Given a total of 519 subjects and 53 years of included age, an even distribution of subjects (dotted line) would include 9.8 subjects for each inclusive year of age. Based on Chi-square goodness of fit analysis, participation was not evenly distributed.

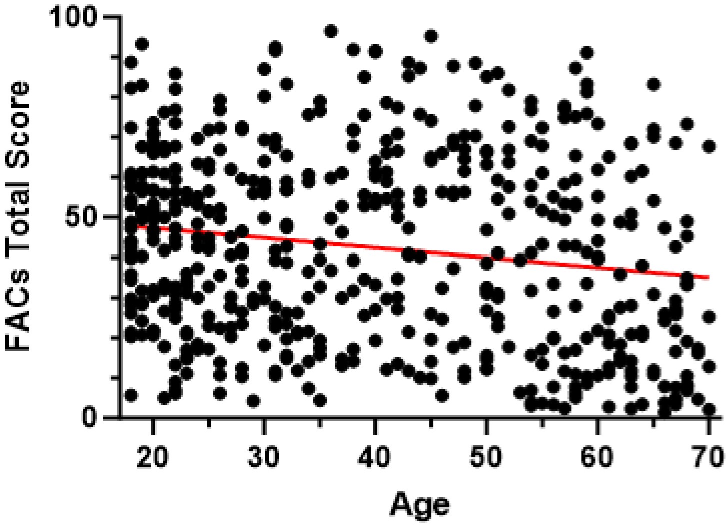

Fig. S2 Age distribution of Total Score for the Fatigue and Altered Cognition Scale (FACs) in an online study of adult participants (18-70

years). Pearson correlation suggests that overall Total Score declined with age (p=0.0001, r= -0.1681).

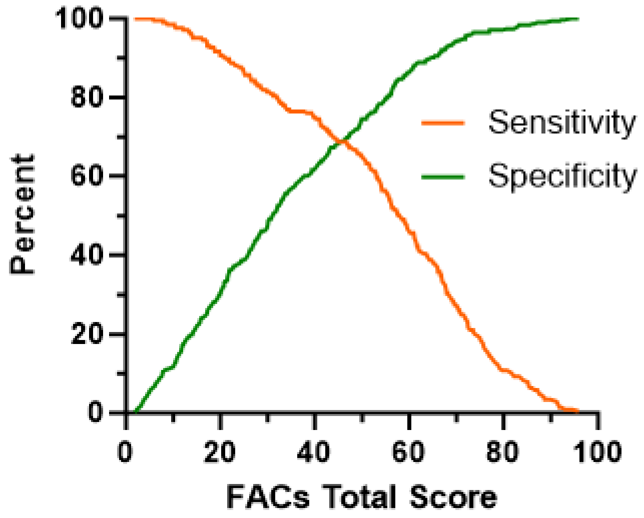

Fig. S3 Individualized sensitivity and specificity plots for the receiver operating characteristics (ROC) curve comparing the Fatigue and Altered Cognition Scale (FACs) questionnaire Total Score between individuals with a history of TBI and no history of TBI.

Survey Question Flow: Screening, Demographics, and Study Questions For the Fatigue and Altered Cognition Scale (FACs)

Start of Block: Screening Questions

Thank you for your interest in this study. Please answer the following screening questions to determine if you are eligible to participate.

Thank you.

Have you been determined **legally incapable** of making decisions on your own behalf?

○ Yes

○ No

Skip To: End of Survey If Have you been determined legally incapable of making decisions on your own behalf? = Yes

What is your age? (please type in)

____

Skip To: End of Survey If Condition: What is your age? (please t... Is Less Than or Equal to 17. Skip To: End of Survey.
Skip To: End of Survey If Condition: What is your age? (please t... Is Greater Than or Equal to 71. Skip To: End of Survey.

Have you had a TBI/concussion?

○ Yes

○ No

End of Block: Screening Questions

Start of Block: Demographics

What is your sex?

- ○ Male
- ○ Female
- ○ Not Listed: Please Specify

Please specify.

____

Please describe your racial/ethnic identity (select all that apply):

- ▢ American Indian or Alaskan Native
- ▢ Asian
- ▢ Black or African American
- ▢ Hispanic or Latino
- ▢ Native Hawaiian or Other Pacific Islander
- ▢ White
- ▢ Biracial/multiracial

Have you been diagnosed with COVID-19?

- ○ Yes
- ○ No

Display This Question:
If Have you been diagnosed with COVID-19? = Yes

When were you diagnosed (month/year)?

End of Block: Demographics

Start of Block: FACs

Please indicate the degree to which each item was true for you **during the past 2 weeks** by marking the line below, ranging from “not at all” to “extremely”.

Not at all **Extremely**

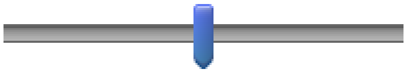

During the past 2 weeks:

Q1/20 - **I felt fatigued**
Q2/20 - **I felt alert**
Q3/20 - **I lost track of what I was going to say**
Q4/20 - **I was forgetful**
Q5/20 - **I had trouble concentrating**
Q6/20 - **I felt worn out**
Q7/20 - **I felt sluggish**
Q8/20 - **I felt run down**
Q9/20 - **I had trouble focusing on things I wanted to do**
Q10/20 - **I had the energy to do what I wanted to do**
Q11/20 - **I was easily confused**
Q12/20 - **I felt "spaced out" like I was in a fog**
Q13/20 - **I had to force myself to get things done**
Q14/20 - **I was clear-headed**
Q15/20 - **I felt tired**
Q16/20 - **I didn’t process things as quickly or accurately as I should have**
Q17/20 - **I had to struggle to finish what I started to do**
Q18/20 - **I had trouble paying attention**
Q19/20 - **It was hard for me to make up my mind and reach a decision**
Q20/20 - **I had problems feeling energetic no matter if I slept or napped**

If you have experienced none of these symptoms, please skip the next question.

How long after your last brain injury did you start to experience any of these problems?

○ Less than 6 months

○ 6 months to 1 year

○ 1 year to 5 years

○ More than 5 years

End of Block: FACs

Start of Block: Ohio State University TBI Identification Method Questionnaire

The following questions are about injuries to your head or neck that you may have had anytime in your life.

In your lifetime, have you ever been hospitalized or treated in an emergency room following an injury to your head or neck? Think about any childhood injuries you remember or were told about.

- ○ Yes
- ○ No

In your lifetime, have you ever injured your head or neck in a car accident or from crashing some other moving vehicle like a bicycle, motorcycle, or ATV?

- ○ Yes
- ○ No

In your lifetime, have you ever injured your head or neck in a fall or from being hit by something (for example, falling from a bike or horse, rollerblading, falling on ice, being hit with a rock)? Have you ever injured your head or neck playing sports or on the playground?

- ○ Yes
- ○ No

In your lifetime, have you ever injured your head or neck in a fight, from being hit by someone, or from being shaken violently? Have you ever been shot in the head?

- ○ Yes
- ○ No

In your lifetime, have you ever been nearby when an explosion or a blast occurred? If you served in the military, think about any combat- or training-related incidents.

- ○ Yes
- ○ No

Think about your most severe head or neck injury.

Were you knocked out or did you lose consciousness (LOC)?

- Yes
- No

Display This Question:
If Were you knocked out or did you lose consciousness (LOC)? = Yes

Approximately how long did the loss of consciousness last?

- Less than 30 minutes
- 30 minutes to 24 hours
- Over 24 hours

Display This Question:
If Were you knocked out or did you lose consciousness (LOC)? = No

Were you dazed or did you have a gap in your memory from the injury?

- Yes
- No

How old were you? (in years)

____

Have you ever had a period of time in which you experienced multiple, repeated impacts to your head (e.g. history or abuse, contact sports, military duty)?

- Yes
- No

Display This Question:
If Have you ever had a period of time in which you experienced multiple, repeated impacts to your he... = Yes

Did you typically experience loss of consciousness with these injuries?

- Yes
- No

Display This Question:
If Did you typically experience loss of consciousness with these injuries? = No

Were you typically dazed, or did you have a gap in your memory from these injuries?

- ○ Yes
- ○ No

Display This Question:
If Did you typically experience loss of consciousness with these injuries? = Yes

Approximately how long did the loss of consciousness last?

- ○ Less than 30 minutes
- ○ 30 minutes to 24 hours
- ○ Over 24 hours

Display This Question:
If Did you typically experience loss of consciousness with these injuries? , Yes Is Displayed

What was the most severe effect from one of the times you had an impact to the head?

____

Display This Question:
If Did you typically experience loss of consciousness with these injuries? , Yes Is Displayed

How old were you when these repeated injuries began? (in years)

____

Display This Question:
If Did you typically experience loss of consciousness with these injuries? , Yes Is Displayed

How old were you when these repeated injuries ended? (in years)

____

End of Block: Ohio State University TBI Identification Method Questionnaire
